# Impact of imputation methods on the amount of genetic variation captured by a single-nucleotide polymorphism panel in soybeans

**DOI:** 10.1186/s12859-016-0899-7

**Published:** 2016-02-02

**Authors:** A. Xavier, William M. Muir, Katy M. Rainey

**Affiliations:** Department of Agronomy, Purdue University, Lilly Hall of Life Sciences, 915 W. State St., West Lafayette, Indiana 47907 USA; Department of Animal Science, Purdue University, Lilly Hall of Life Sciences, 915 W. State St., West Lafayette, Indiana 47907 USA

**Keywords:** Empirical Bayes, Heritability, Genomic selection, Association studies

## Abstract

**Background:**

Success in genome-wide association studies and marker-assisted selection depends on good phenotypic and genotypic data. The more complete this data is, the more powerful will be the results of analysis. Nevertheless, there are next-generation technologies that seek to provide genotypic information in spite of great proportions of missing data. The procedures these technologies use to impute genetic data, therefore, greatly affect downstream analyses. This study aims to (1) compare the genetic variance in a single-nucleotide polymorphism panel of soybean with missing data imputed using various methods, (2) evaluate the imputation accuracy and post-imputation quality associated with these methods, and (3) evaluate the impact of imputation method on heritability and the accuracy of genome-wide prediction of soybean traits. The imputation methods we evaluated were as follows: multivariate mixed model, hidden Markov model, logical algorithm, k-nearest neighbor, single value decomposition, and random forest. We used raw genotypes from the SoyNAM project and the following phenotypes: plant height, days to maturity, grain yield, and seed protein composition.

**Results:**

We propose an imputation method based on multivariate mixed models using pedigree information. Our methods comparison indicate that heritability of traits can be affected by the imputation method. Genotypes with missing values imputed with methods that make use of genealogic information can favor genetic analysis of highly polygenic traits, but not genome-wide prediction accuracy. The genotypic matrix captured the highest amount of genetic variance when missing loci were imputed by the method proposed in this paper.

**Conclusions:**

We concluded that hidden Markov models and random forest imputation are more suitable to studies that aim analyses of highly heritable traits while pedigree-based methods can be used to best analyze traits with low heritability. Despite the notable contribution to heritability, advantages in genomic prediction were not observed by changing the imputation method. We identified significant differences across imputation methods in a dataset missing 20 % of the genotypic values. It means that genotypic data from genotyping technologies that provide a high proportion of missing values, such as GBS, should be handled carefully because the imputation method will impact downstream analysis.

## Background

Marker-assisted selection (MAS) is a powerful tool for accelerating genetic improvement of plants and animals [1] through introgression of quantitative trait loci (QTL) and selection using genomic-enhanced breeding values (GEBV) [18, 27]. Meuwissen et al. [31] and Xu [64] first incorporated genome-wide molecular markers into the estimation of breeding values, and these so-called genomic selection (GS) methods have improved over time to handle a large number of loci [24, 59] with increased accuracy [7].

For genetic improvement and genetic studies in the post-genomic era, new genotyping platforms such as genotyping by sequencing (GBS) [10] and single-nucleotide polymorphism (SNP) arrays [45] provide a large number of molecular markers, but often with reduced genotyping accuracy [16, 52] and missing genotypes due to other factors. The implementation of such technology in crop breeding programs [18, 22, 48] helps maximize genetic gain through genomic selection [27, 56, 57] and also benefits genome wide association studies (GWAS) [17, 37, 52]. When there is a high proportion of missing data, consistent imputation may be challenging [22, 42, 53]. Accurate imputation of missing values and correction of genotyping errors is essential for eliminating gaps in genome coverage, integrating data across different arrays, and allowing robust genome-wide association mapping and prediction [21, 30, 49].

Researchers have proposed a variety of procedures for adjusting genotypic data to impute missing values and correct SNP miscalls. Most imputation algorithms, such as Hidden Markov Models [46], linear models [8, 65] and pedigree-based haplotyping [58], were designed for ordered markers which are known because there is a reference genome available. But some methods of imputation do not require information on marker order or phase [17, 44] such as k-Nearest Neighbors [55], Single Value Decomposition [36], or Random Forest [50]. These, therefore, are well-suited for *de novo* genotyping [3, 43].

Incorporating genealogical information boosts the accuracy of genotypic imputation, phasing, and the power of association analysis [16, 23]. Therefore, here we describe a new method of imputation based on the pedigree relationship matrix [62] and covariance among markers. The proposed method treats genotypes as response variables within a multivariate empirical Bayes model traditionally used in plant and animal breeding [19]. The expectation of narrow-sense heritability across a set of traits can be translated into a machine learning approach of pattern recognition to measure the amount of additive genetic information captured by the genomic information. Thus, we then compare the accuracy of each imputation method based on the amount of genetic variation that can be accounted for by a SNP panel [16].

## Methods

This study employed genotypes and phenotypes from the SoyNAM project (soynam.org), downloaded on September 10^th^, 2014. SoyNAM is a soybean (*Glycine max*, Merr.) maturity group III nested association panel composed of 40 bi-parental crosses sharing a common parent and inbred for five generations. The panel comprises 5,596 recombinant inbred lines, phenotyped in 18 combinations of year and location, for four agronomic traits: grain yield, plant height, days to maturity, and seed protein composition. The panel was genotyped with a designed 5 k SNP chip. After removing non-segregating markers, low quality SNPs based on marker heritability (0.99) [11] and minor allele frequency (0.05) [54], we selected a set of 4,246 SNPs for this study that had 1 % of its genotypic data missing. To compare imputation performance under conditions with more missing data, we generated a second dataset by randomly deleting 20 % of genotypic data across all individuals using the prodNA function from the R package missForest [50]. If differences in imputation methods are detectable for a strict amount of 20 % missing values, these results will apply to datasets with a larger proportion of missing values.

We compared imputation approaches using a variety of methods: a multivariate mixed model (MMM); Hidden Markov Models (HMM) implemented in three software packages commonly employed in genetic studies [30]; a logical algorithm; and three non-parametric methods k-Nearest Neighbor (kNN), Single Value Decomposition (SVD), and Random Forest (RF). Raw and imputed datasets are available upon request.

### Imputation methods

#### Multivariate mixed model

We adapted the multivariate mixed model method for imputation from Gengler et al. [13] and Yang et al. [65], based on the concept that marker inheritance will proceed in a Mendelian manner and therefore, follow the pedigree [41]. The method bases the numerator relationship matrix on the expectation of Mendelian allele inheritance with shared identity by descent (IBD). This contrasts with expectation-maximization imputation algorithms that rely on observed kinship [47, 65] and imputation through coalescent analysis [23] that attempts to recreate the pedigree.

If allele inheritance follows the pedigree, the marker should be perfectly heritable, except for Mendelian sampling error. Consequently, SNP heritability is an indicator of the gene content [11, 13], and we can estimate it by fitting the marker to a mixed model with pedigree specified as a random effect, as discussed by Forneris et al. [11]. Thus, missing SNPs can be imputed as empirical Bayes estimates. Expanding the model to a multivariate level further improves the accuracy of prediction, “borrowing strength” from flanking markers and related individuals [16]. All markers are evaluated in a panel in a sliding window across the genome, ordered according to the genetic or physical map [9]. The method considers the marker of interest (**y**_j_) and its flanking markers (**y**_i_ and **y**_k_) as response variables (**Y** = {**y**_i_, **y**_j_, **y**_k_}), coding them in an ordinal or continuous scale [51], and fitting them to a multivariate mixed model. Thus:

$$ {\mathbf{Y}}_{\mathrm{n}\times 3}=1{\boldsymbol{\upmu}}_{\mathrm{n}\mathrm{x}3}+{\mathbf{Z}}_{\mathrm{n}\times \mathrm{n}}{\boldsymbol{\upgamma}}_{\mathrm{n}\times 3}+{\boldsymbol{\upvarepsilon}}_{\mathrm{n}\times 3} $$

where **Z** is the design matrices of the random effects, **μ** and **γ** represent the intercept and individual additive effect, and **ε** represents the residual term. The variances associated with the imputed marker are: ${\upsigma}_{\upgamma_{\mathrm{j}}}^2$, representing the genetic variance of the marker j; σ_ij_, the genetic covariance between marker j and the flanking marker i; σ_ik_, the genetic covariance between marker j the flanking marker k; and ${\upsigma}_{\upvarepsilon_{\mathrm{j}}}^2$, the residual variance of marker j. For the given mode, variances are expressed as

$$ \mathrm{V}\mathrm{a}\mathrm{r}\left(\mathbf{Y}\right)=\left(\mathbf{A}\otimes {\upsigma}_{\upgamma}^2+\mathbf{I}\otimes {\upsigma}_{\upvarepsilon}^2\right)=\left(\mathbf{A}\times \left[\begin{array}{ccc}\hfill {\upsigma}_{\upgamma_{\mathrm{i}}}^2\hfill & \hfill {\upsigma}_{\upgamma_{\mathrm{i}\mathrm{j}}}\hfill & \hfill {\upsigma}_{\upgamma_{\mathrm{i}\mathrm{k}}}\hfill \\ {}\hfill {\upsigma}_{\upgamma_{\mathrm{i}\mathrm{j}}}\hfill & \hfill {\upsigma}_{\upgamma_{\mathrm{j}}}^2\hfill & \hfill {\upsigma}_{\upgamma_{\mathrm{j}\mathrm{k}}}\hfill \\ {}\hfill {\upsigma}_{\upgamma_{\mathrm{i}\mathrm{k}}}\hfill & \hfill {\upsigma}_{\upgamma_{\mathrm{j}\mathrm{k}}}\hfill & \hfill {\upsigma}_{\upgamma_{\mathrm{k}}}^2\hfill \end{array}\right]+\mathbf{I}\times \left[\begin{array}{ccc}\hfill {\upsigma}_{\upvarepsilon_{\mathrm{i}}}^2\hfill & \hfill 0\hfill & \hfill 0\hfill \\ {}\hfill 0\hfill & \hfill {\upsigma}_{\upvarepsilon_{\mathrm{j}}}^2\hfill & \hfill 0\hfill \\ {}\hfill 0\hfill & \hfill 0\hfill & \hfill {\upsigma}_{\upvarepsilon_{\mathrm{k}}}^2\hfill \end{array}\right]\ \right) $$

where **A** is the additive numerator relationship matrix and **I** is an identity matrix. MMM uses restricted maximum likelihood (REML) to estimate genetic covariances and then replaces missing values for the central position by the multivariate empirical Bayes estimate [66], also referred to as the best linear unbiased predictor (BLUP) [19]. The model is then incremented to the next position and repeated.

This study used the software BLUPF90 [32, 33] to compute the covariance components. However, any existing software that allows multivariate mixed models incorporating pedigree information can implement the model, such as Wombat, ASReml, or SAS. Efficient algorithms to compute mixed models are described by Zhou and Stephens [66], Legarra and Misztal [24] and VanRaden [59].

#### Hidden Markov models

The HMM method is commonly employed in genetics and genomics for stochastic modeling of Markov processes (such as the computation of haplotypes). Assuming ordered markers, the HMM estimates the most likely path of states (i.e. genotype) based on the transition probability of marker M^t^ to change state given the previous marker M^t − 1^. In genetic terms, the four possible states for a diploid organism with alleles M_1_ and M_2_ for locus M, are: M_1_M_1_, M_1_M_2_, M_2_M_1_ and M_2_M_2_.

This study evaluated three HMM software programs: fastPHASE [46], Beagle [4], and MaCH [26]. Beagle, fastPHASE, and MaCH implement HMM with iterative updating via Expectation-Maximization (Baum–Welch algorithm). Missing values of each chromosome were imputed separately and pedigree information was not provided. HMM is the most common method of imputation and is shown to boost power and resolution of genome-wide association studies [21, 30].

#### Logical algorithm

This method is implemented in the program findhap.f90 [58]. The program is computationally efficient, suited for large datasets, and becomes increasingly accurate as the proportion of pedigree data increases [38]. Thus it is advantageous for populations that pursue genotyped pedigrees.

Findhap.f90 first generates a list of possible haplotypes, then adds a genotype into the haplotype list and searches for a matching haplotype, then finds the second haplotype by subtracting the first haplotype from the genotype. It then compares each genotype to the haplotype list and imputes unknown alleles from the haplotypes. The first two iterations use population-wide genotypic data, and subsequent iterations locate matching haplotypes from pedigree data [57].

#### k-Nearest neighbor

kNN is a non-parametric method commonly used for prediction and classification. kNN is a memory-based learning algorithm based on voting [61]. It relies on filling missing data points with the weighted mean of the k most similar genotypes based on Euclidean distance (root sum-of-squares of differences) between standardized observations. Rutkoski et al. [44] evaluated kNN for genotypic imputation and suggested it was a promising method. We used the package knnGarden [60] with a setting of k = 10 to perform our computation.

#### Single value decomposition

SVD relies on orthogonal expression of the genotypic matrix [55] from the decomposition **M** = **UDV**^T^. SVD uses the most significant eigenvectors (columns of **U**) to predict missing values. We performed SVD imputations by chromosome using the bi-cross-validation algorithm described in Owen & Perry [36] and implemented in package bcv [40].

#### Random forest

Random Forest [2] is a non-parametric method of prediction, classification, and imputation of mixed data types [50]. It establishes a combination of decision tree predictors, where the trees are bootstrapped random independent vectors that constitute training forests [2]. Imputation studies of GBS data in wheat breeding have reported promising results for RF [42–44]. We performed imputations employing random forest by chromosome to generate more informative trees and reduce computational burden, using the package missForest [50].

### Comparison of methods

Imputation errors impact the reliability of breeding values [6, 43] and therefore, affect genome-wide selection. To evaluate the fitness of different imputation methods for genomic enhanced breeding values (GEBV), we calculated the intra-class correlation coefficient (ICC) for the genetic parameter, generally known as heritability, through average information restricted maximum likelihood, implemented in AI-REMLF90 with a convergence criterion of 10^-11^ [33]. The ICC is defined as

$$ {\mathrm{h}}^2=\frac{\upsigma_{\mathrm{A}}^2}{\upsigma_{\mathrm{Y}}^2} $$

given the phenotypic variance σ_Y_^2^ = σ_A_^2^ + σ_E_^2^ + σ_C_^2^ + σ_ε_^2^; where σ_A_^2^ is the additive genetic variance, σ_E_^2^ is the variance due to environment, σ_C_^2^ is the variance due to microenvironment (i.e. controls), and σ_ε_^2^ is the residual variance. In genetic terms, ICC represents narrow-sense heritability, which is defined as the amount of genetic variation that alleles can transmit to the following generation, and it quantifies the population response to selection [28]. The rational for using narrow-sense heritability estimator as a measure of how well genetic variability is captured by the genotypic information comes from its practical application of pattern recognition in machine learning [15, 34]. Genomic relation matrices generated from a SNP panel imputed through distinct methods would yield different values of variance components. Consequently, kernels that provide enhanced genetic variance and higher heritability reflect better estimators of the genetic term [35].

**Fig. 1:**
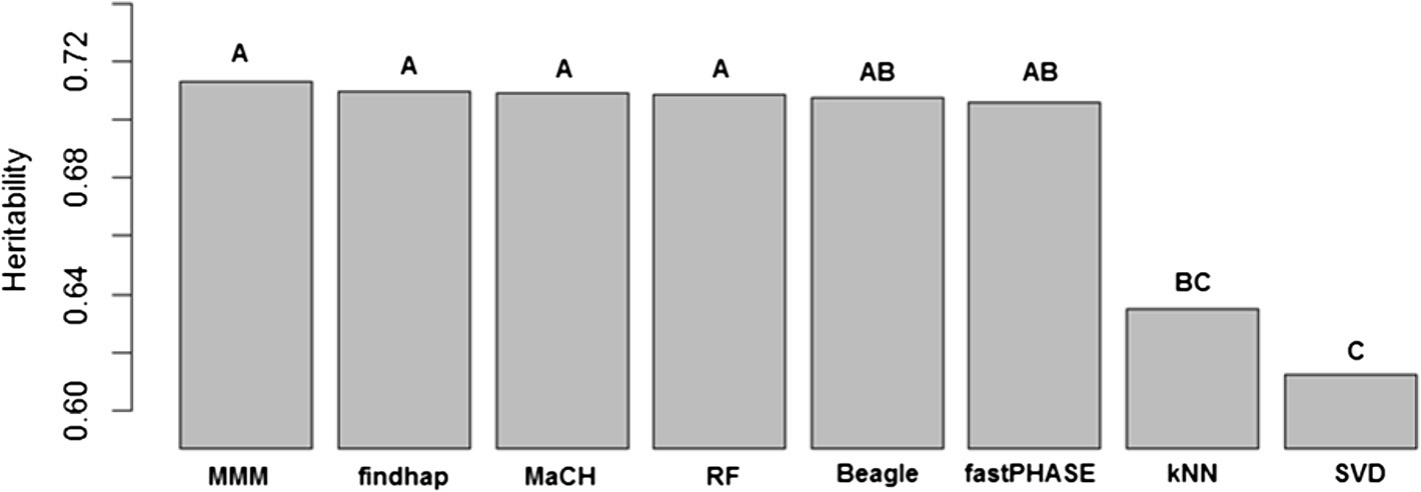
Average heritability across four soybean traits using genotypic data imputed with different methods. Letters represent the statistical difference based on Tukey’s HSD procedure (α = 0.05)

To compare the effect of imputation methods on heritability, we performed analysis of variance (ANOVA) following the linear model y_ijkl_ = μ_._ + τ_i_ + β_j_ + γ_k_ + ε_ijkl_; where y_ijkl_ is the heritability of the i^th^ imputation method (i = 1, …, 8) with j^th^ percentage of missing loci (j = 1, 20) of the k^th^ trait (k = 1, …, 4) in the l^th^ observation; μ_._ is the overall mean; τ_i_ represents the imputation method; β_j_ is the percentage of missing loci; γ_k_ represents the trait; and ε_ijkl_ is the residual of the ijkl observation. In this model, imputation method is the parameter of interest while blocking trait and percentage of missing. Levene’s test determined the equality of variances [12]. The Shapiro-Wilk test of normality verified the normality. Tukey’s honest significant difference (HSD) (α = 0.05) grouped the imputation methods when the *p*-value of F test was significant (<0.05). Results shown in Fig. 1.

### Imputation accuracy and prediction accuracy

This study evaluated imputation accuracy by determining the proportion of data imputed that was identical to the standard dataset [56]. For each method, we compared the imputed genotypic matrix with 20 % of the loci missing to the genotypic matrix corrected using the same method with 1 % of loci missing. Thus, the level of accuracy provides insight into both imputation method and modifications of the dataset.

To measure prediction accuracy in the context of genomic selection, we used the correlation of observed and predicted values in a five-fold cross validation divided by the square root of the heritability as described by Lehermeier et al. [25]. We used the following whole-genome regression methods [7, 14] to generate the predicted values: BayesA, BayesB, BayesCπ, Bayesian LASSO, and Bayesian Ridge Regression, as implemented by Pérez and de los Campos [39] with default settings of hyperpriors. The statistical testing of imputation method on prediction accuracy followed the same ANOVA model previously described with an additional term to accommodate the whole-genome regression method.

## Results

### Effect of imputation method on heritability

We found a significant association (*p*-value < 0.01) between heritability values (y_ijkl_) and all terms in the statistical model: imputation method (τ_i_), percentage of missing values (β_j_) and agronomic trait (γ_k_). The fitted model provided a coefficient of determination R^2^ = 0.95 and a coefficient of variation CV = 6.72. The group means procedure (Fig. 1) showed that most imputation methods were similar. MMM had provided the highest across-trait heritability, although not significantly better than the other methods except for the kNN and SVD methods, which were inferior to the others, with SVD being the worst.

### Post-imputation quality parameters

**Fig. 2:**
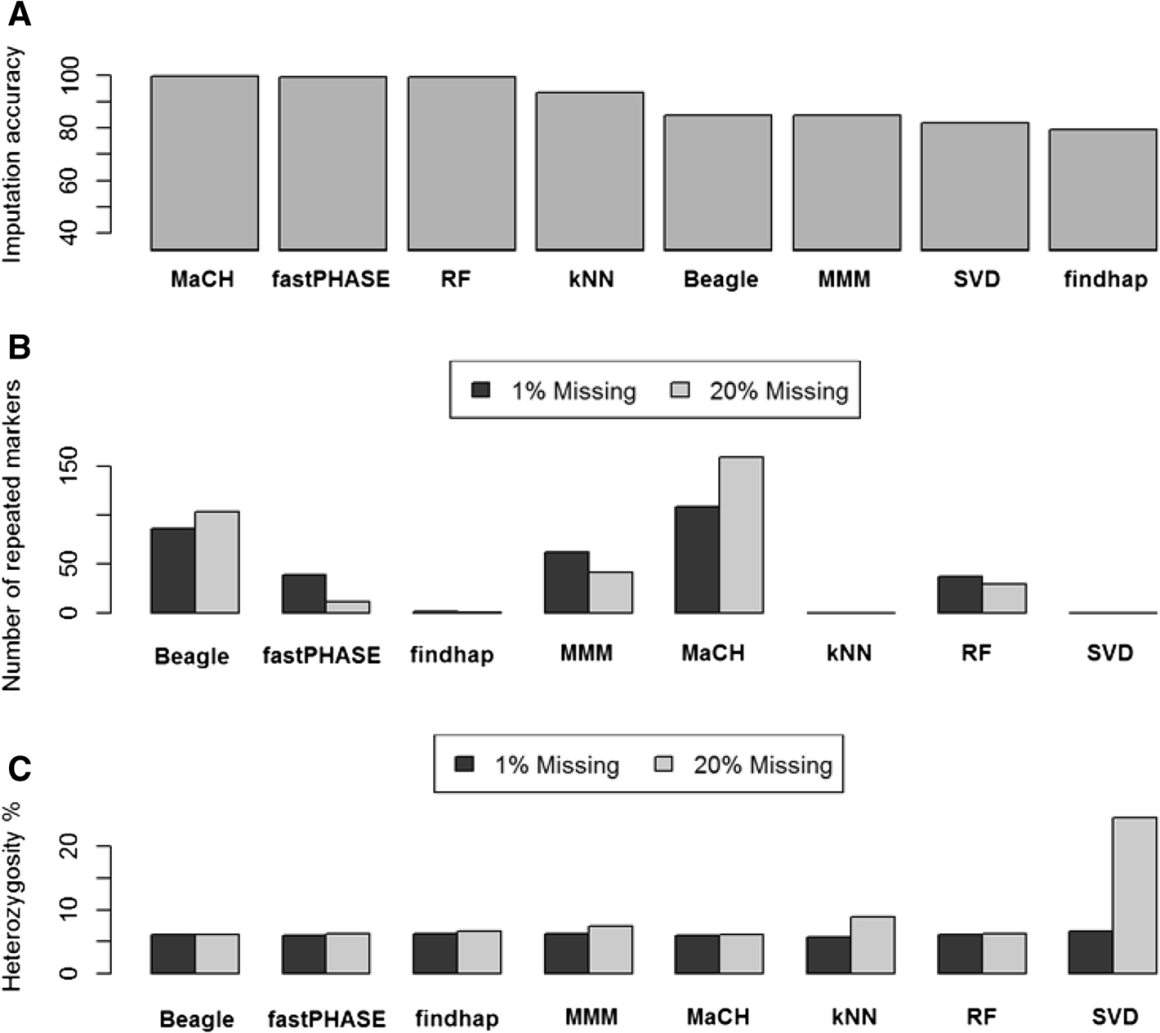
Post-imputation quality parameters. **a** Imputation accuracy measured as the percentage of identical loci between datasets with 1 and 20 % missing. **b** Number of repeated markers (ie. full LD) after imputations with 1 and 20 % missing data. **c** Percentage of heterozygous loci after imputations of datasets with 1 and 20 % missing data

The average accuracy of imputation for data ranged from 79.32 to 99.60 %, (Fig. 2a). MaCH, fastPHASE, and RF were nearly identical (>99.5 %), followed by kNN (93.5 %). MMM and Beagle showed the same performance (85 %). Imputation accuracy reflects the sensitivity of imputation methods to missing data. However, lower imputation accuracy can be attributed to more severe correction of genotyping errors (e.g. SNP miscalls).

We observed a higher number of markers with full linkage disequilibrium (i.e. repeated markers) after imputation with the HMM implementations. MaCH and Beagle (Fig. 2b) also increased the number of repeated markers as the percentage of missing data increased. Non-parametric methods, SVD and kNN, did not provide repeated markers. Random forest and both pedigree-based methods, MMM and findhap, showed fewer repeated markers as the missing data increased.

The third measure of accuracy of imputation and data correction is the difference between the observed and expected proportion of heterozygosity in the data. The expected proportion of heterozygosity in this dataset is 6.25 % of loci given that the parents were homozygous and the plants were genotyped in the F5 generation (i.e. after 5 generations of selfing), in which the expected inbreeding coefficient is 93.75 %. The results show that the proportion of heterozygous loci tends to increase as the proportion of missing data increases (Fig. 2c). We observed this in all but two methods, Beagle and MaCH. SVD and kNN were more likely to increase the number of heterozygous loci with more missing data, especially SVD for which proportions of heterozygosity reached 24 %, meaning that heterozygous loci were assigned to most missing loci.

### Imputation methods and genetic variation captured

**Fig. 3:**
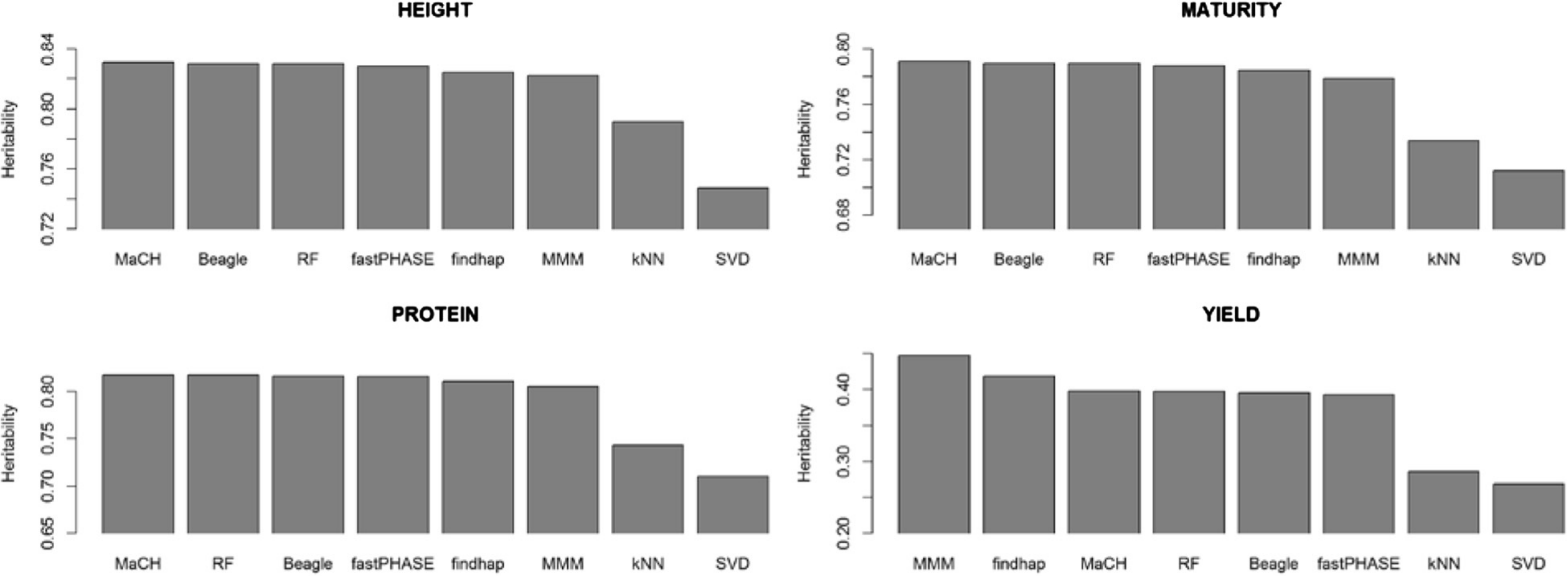
Narrow-sense heritability for soybean agronomic traits computed for genotypic datasets imputed by various methods

The mean heritability for traits in this study was 0.81 ± 0.04 for plant height, 0.77 ± 0.04 for days to maturity, 0.79 ± 0.06 for seed protein content, and 0.37 ± 0.08 for grain yield. Grain yield is considered minimally heritable while the other three are highly heritable. Imputation method had the greatest impact on the trait with the lowest heritability (grain yield) when compared to the impact on traits with high heritability (Fig. 3). The MMM method was superior to the other methods for minimally heritable traits while MaCH was best for highly heritable traits. The SVD and kNN methods were consistently inferior across traits.

The prediction accuracy across traits ranged from 0.606 (MMM) to 0.628 (RF), but did not differ significantly (*p*-value = 0.537). However, it appears that the genotypic data imputed with RF may have a slightly better performance. Whole-genome regression methods did not provide statistically significant differences in prediction accuracy (data not shown).

## Discussion

### The capture of genetic variation

Differences between imputation methods were clearly detectable on a SNP panel containing 20 % missing values, raising questions about the quality and reliability of genotypic data obtained by imputing datasets with an 80 % or greater proportion of missing values, a common scenario for GBS.

Our results indicate that genotypes imputed with methods that rely on pedigree information, such as MMM, better capture genetic variance in complex low heritability traits, while imputations performed using HMM may favor traits with high heritability. The choice of imputation method will affect downstream analysis. Thus, if the genetic architecture of the traits to be analyzed is known, it is possible to achieve more accurate results by selecting the most suitable imputation method.

The genetic architecture of the trait should also influence the choice of genomic prediction method [7], although this study found no statistically significant differences, likely due to the similar nature of evaluated models also reported by Howard et al. [20]. Chen et al. [5] and Poland et al. [43] reported interactions between imputation method and prediction accuracy for different traits. For example, genotypic imputation through SVD showed inferior capture of genetic variance, but it did not affect prediction accuracy.

### MMM: strengths and weaknesses

In this study we presented a mixed model method of imputation using pedigree information that displayed interesting properties regarding the capture of genetic variance and may have advantages for downstream analysis of complex traits. Some reported advantages of MMM include imputation of un-genotyped individuals [41] or with a large percentage of missing data [65] without losing robustness; and the identity link function of MMM allows imputation regardless of the allelic coding [51].

On the other hand, genotypic information imputed by MMM was not advantageous for analyzing highly heritable traits and it did not improve prediction accuracy. However, NAM populations have a very simple pedigree structure and more complex pedigree structures may lead to better results [29]. Another drawback was the method’s computational burden because the mixed model computes each molecular marker, a limitation that can be addressed through parallel computing.

Much as in HMM, the change-of-state of a haplotype in MMM is controlled by the covariance, with flanking markers considering linkage disequilibrium (LD), and the population structure allied to the pedigree information simultaneously, while forward-backward HMM algorithms work one direction at time using just a subset of random samples [3]. Imputation methods that incorporate pedigree do not rely on samples from a reference dataset that is assumed to comprise all populations [16]. Using the genomic relationship instead of pedigree, Yang et al. [65] have reported the superiority of a similar MMM method over HMM emphasizing better incorporation of information on LD and IBD.

### Choice of the imputation method

Properties of the methods are summarized in Table 1. Our results support the use of HMM and RF for a reliable representation of the genotypes, being best for analyses of highly heritable traits and a good alternative even if pedigree data are not available. In addition, imputation though MMM and findhap is preferred to analyze traits with low heritability when the pedigree is known. kNN and SVD provided consistently poor representations of the genotypes.

**Table 1:** Summary of properties of imputation methods under evaluation

Method	Better fit for low heritability traits	High imputation accuracy	Enlarges LD blocks	Corrects Miscalls	Accommodates pedigree information	Suitable for unordered markers
Beagle			X	X	X	
fastPHASE	X	X		X		
findhap					X	X
kNN						X
MaCH		X	X	X	X	
MMM	X			X	X	
RF		X				X
SVD						X

For GBS and other technologies where a high percentage of missing values is expected, our results support the use of MaCH, fastPHASE, and RF, methods that have shown insensitivity to the percentage of missing loci. In a similar study, [17]) observed inferior performance of RF over HMM in wheat that could be attributed to the whole-genome imputation at once as opposed to one chromosome at a time, resulting into less informative decision trees and inconsistent imputation. The imputation of each chromosome separately is also a common practice with HMM [29].

## Conclusions

Quality of the imputation disturbs the genetic variance captured by the genotypic data. We were able to show that the imputation of genotypic data in the case where proportion of missing values as low as 20 % can affect the quality of the genotypic representation by the SNP panel. This results must be seen as a word of care for technologies based on low-coverage genotyping, such as GBS, where the amount of missing information commonly achieves values of 80 %. Yet, for the scenario in study it was not possible to identify significant impact of imputation method on genomic prediction. Thus, based on imputation accuracy and genetic variance captured by the SNP panel, the imputation method choice is hidden Markov models and random forest for general analysis. Pedigree-based methods of imputation were recognized to enhance the heritability of grain yield, the lowest heritable trait in this study.

### Availability of supporting data

This study employed genotypes and phenotypes from the SoyNAM project (soynam.org). Data is available through the R package SoyNAM [63] and USDA-ARS soybean database SoyBase (soybase.org).
